# Homogenized Finite Element Analysis on Effective Elastoplastic Mechanical Behaviors of Composite with Imperfect Interfaces

**DOI:** 10.3390/ijms151223389

**Published:** 2014-12-16

**Authors:** Wu-Gui Jiang, Ren-Zhi Zhong, Qing H. Qin, Yong-Gang Tong

**Affiliations:** 1 School of Aeronautical Manufacturing Engineering, Nanchang Hangkong University, Nanchang 330063, China; E-Mail: lukerenzhi@126.com; 2 Research School of Engineering, the Australian National University, Acton, ACT 2601, Australia; E-Mails: qinghua.qin@anu.edu.au (Q.H.Q.); tygiaarh419@163.com (Y.-G.T.)

**Keywords:** fiber-reinforced ceramic matrix composites, homogenization, interface effect, tensile strength, finite element method

## Abstract

A three-dimensional (3D) representative volume element (RVE) model was developed for analyzing effective mechanical behavior of fiber-reinforced ceramic matrix composites with imperfect interfaces. In the model, the fiber is assumed to be perfectly elastic until its tensile strength, and the ceramic material is modeled by an elasto-plastic Drucker-Prager constitutive law. The RVE model is then used to study the elastic properties and the tensile strength of composites with imperfect interfaces and validated through experiments. The imperfect interfaces between the fiber and the matrix are taken into account by introducing some cohesive contact surfaces. The influences of the interface on the elastic constants and the tensile strengths are examined through these interface models.

## 1. Introduction

As an important type of ceramic matrix composites, fiber-reinforced ceramics (FRCs) such as carbon-fiber/silicon-carbide (C/SiC) are becoming popular and important due to their unique thermal, mechanical and chemical stability in various environments, high strength and excellent thermal shock resistance of ceramics, and high toughness of carbon fibers at elevated temperature [1]. The assessment of mechanical properties of such composites is, however, much more complex than that of conventional ceramics, as the composites may be partly or highly anisotropic. Usually, the physical and mechanical properties of FRCs depend on properties of their constituents and the corresponding geometry and concentration (e.g., volume fraction of fibers, fiber/matrix interphase structure, fiber weave architecture, and matrix properties). It is noted that when the fibers are embedded into the ceramic matrix to form composites, the matrix bonds fibers together and transfers loads to the fibers through the interfaces between them. Thus, the fiber/matrix interfaces govern to some extent the transverse tensile strength and the fracture behavior of the composite [2]. Experimental studies [3,4] revealed that interfacial properties also play an important role in affecting the macroscopically effective properties of FRCs.

Existing schemes for predicting macroscopically effective properties of composites include Mori-Tanaka method [5,6], self-consistent method [7,8], generalized self-consistent method [9], combination of the Mori-Tanaka method and the iso-stress or iso-strain assumptions [10], Christensen’s approach [11], and various mathematical homogenization methods [12,13]. Many works, for example [14,15,16], have been done to study the effects of interfacial properties on effective properties of composites, but the components of composites were usually assumed to be elastic for simplicity in the most existing theoretical models. Ju and Yanase [17] proposed an elasto-plastic damage formulation to predict the overall transverse mechanical behavior of continuous fiber reinforced ductile matrix composites with the framework of micromechanics and homogenization by incorporating the interfacial damage. Alternatively, through investigating interphase effect on elastic and thermal conductivity response of polymer composite materials, Mortazavi *et al.* [18,19] compared the capability of the Mori-Tanaka method and the three-dimensional (3D) finite element (FE) analysis and concluded that despite complexities for modeling of high volume concentrations and aspect ratios for fillers, FE simulations are more reliable and promising than the other schemes. Based on FE method, Taliercio and Coruzzi [20] estimated in-plane transverse strengths using a representative volume element (RVE), in which the perfect bonding is assumed. Yang and Qin [21,22] investigated effective elastic-plastic properties of fiber-reinforced composites. Caporale *et al.* [23] implemented an interfacial failure model by connecting the fibers and the matrix at the finite element nodes by normal and tangential brittle-elastic springs, in which the matrix and fibers are considered homogeneous, isotropic and linearly elastic. Rahul-Kumar *et al.* [24] concluded that the cohesive element can be used to describe the polymer interfacial fracture. These works did not, however, couple the brittle material constitutive law and interfacial debonding in the approaches mentioned above. In addition, those models are not easy to be realized in practical analysis on the effect of interfacial properties on the macroscopically effective elastoplastic properties of composites.

The purpose of this study is to develop a 3D RVE model based on a unidirectional, long-fiber-reinforced ceramic matrix composites, using the computational homogenization FE method which can handle imperfect interface between the fiber and the matrix. Then, the model is incorporated into the commercial FE software ABAQUS through a user subroutine interface. In the RVE, the fiber is assumed to be linear elastic before the stress reaches its tensile strength and the ceramic material is modeled by an elasto-plastic Drucker-Prager constitutive law. The imperfect interfaces between fiber and matrix are taken into account by introducing some cohesive contact surfaces. Making use of the proposed model, comprehensive analyses on the influence of interfacial properties on the macroscopically effective elasto-plastic properties of composites, including the macroscopic stiffness and strength are conducted.

## 2. Results and Discussion

### 2.1. Model Validation

The reliability of both the present periodic boundary condition (PBC) and homogeneous boundary conditions (HPC) models is first assessed in estimating the effective elastic constants of FRCs by comparing them with theoretical results. The macroscopic elastic constants of the composites obtained using the present PBCs and HBCs models are depicted in Table 1. For comparison, the overall properties estimated using the Mori-Tanaka method [6,25], the self-consistent method [7,26] and the modified self-consistent method [8], are also calculated here and listed in Table 1. It can be seem from Table 1 that results from the present model show a good agreement with the theoretical results.

**Table 1:** Comparison of the present PBC and HBC models with some other theoretical solutions.

Models	*E*_1_ (GPa)	*E*_3_ (GPa)	*G*_12_ (GPa)	*G*_23_ (GPa)	*v*_23_
Present PBC model	392.0	391.0	164.9	165.6	0.179
Present HBC model	391.1	393.5	167.6	173.5	0.174
Mori-Tanaka’s method [6,25]	391.7	391.0	165.6	165.1	0.179
Self-consistent method [7,26]	391.6	391.0	165.5	165.2	0.180
Modified self-consistent method [8]	386.6	389.0	161.6	165.6	0.179

### 2.2. Influence of the Interfacial Properties on the Overall Elastic Properties

In the cohesive model, the interface penalty stiffness *K*_interface_ is defined as a function of the interface thickness, *h*_interface_, and the elastic modulus of the interface, *E*_interface_, *i.e.*, $K_{\text{interface}}=E_{\text{interface}}/h_{\text{interface}}$ We introduce an interfacial stiffness dimensionless parameter $\tilde{k}=K_{\text{interface}}/\left[\left(E_{m}+E_{f}\right)/2h_{\text{interface}}\right]$ to represent relative modulus compared with the average of elastic moduli of matrix and fiber. In all simulations, we assume the interfacial thickness *h*_interface_ as one tenth of the carbon-fiber radius. $\tilde{k}=1$ when *E*_interface_ equals to $\left(E_{m}+E_{f}\right)/2$. *K*_nn_ = *K*_tt_ = *K*_ss_ = *K*_interface_, and the other *K_ij_* (i≠j) are specified as zero. The damage initiation criterion and evolution law are not defined in this subsection because they influence the macroscopic elastic properties slightly in the initial small elastic deformation stage.

**Figure 1:**
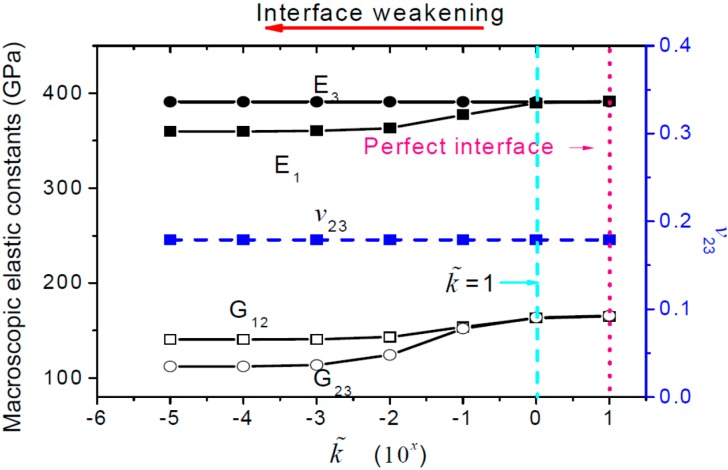
Influence of interface stiffness on the effective elastic constants (the arrows in red and in lightblue represent prefect interface and $\tilde{k}$ = 1, respectively).

**Figure 2:**
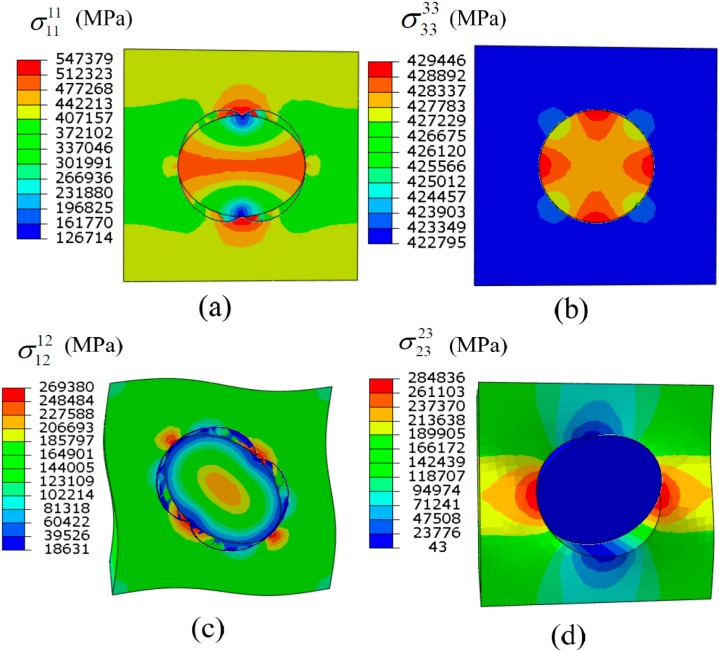
Stress influence functions obtained in the (**a**) tension along the *y*_1_; (**b**) tension along the *y*_3_; (**c**) shear along *y*_1_*y*_2_; and (**d**) shear along *y*_2_*y*_3_ linear perturbation steps in case of $\tilde{k}=1\times 10^{-5}$.

Figure 1 plots the change trends of the macroscopic elastic constants with respect to the interfacial stiffness. It can be seen from Figure 1 that the overall elastic constants *E*_1_, *G*_12_ and *G*_23_ decrease gradually as the interfacial stiffness decreases, but they change slightly when the interface is stronger than the average value of the fiber and the matrix. The longitudinal Young’s modulus *E*_3_ along the fiber direction and *v*_23_ are independent of the interfacial stiffness. We list the contour plots of the stress influence functions obtained in the linear perturbation steps including tension along *y*_1_, tension along *y*_3_, shear along *y*_1_*y*_2_, and shear along *y*_2_*y*_3_ directions in case of $\tilde{k}=1\times 10^{-5}$ in Figure 2, from which it can be found that the bonding of the fiber and the matrix in the composites, subject to the longitudinal tension, will not be affected by the low interfacial stiffness.

### 2.3. Mesh-Sensitive Analysis and Model Validation in Estimating the Ultimate Tensile Strength

**Figure 3:**
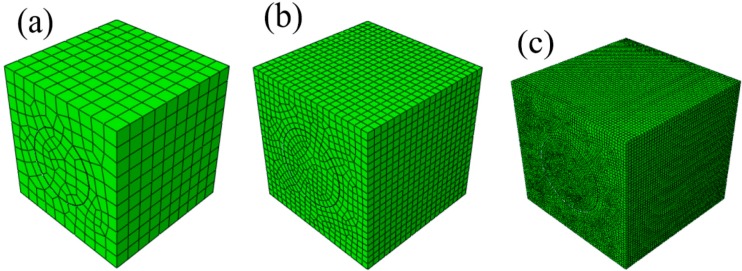
Mesh-sensitivity analysis in the nonlinear analyses: (**a**) coarse; (**b**) medium; and (**c**) fine mesh densities.

Before investigating the ultimate tensile strength, a nonlinear numerical study is first performed to study the sensitivity of the predicted macroscopic responses of the considered FRCs to mesh refinement. Three mesh densities for the unit cell are considered, namely a “coarse”, a “medium” and a “fine” mesh, as shown in Figure 3.

**Figure 4:**
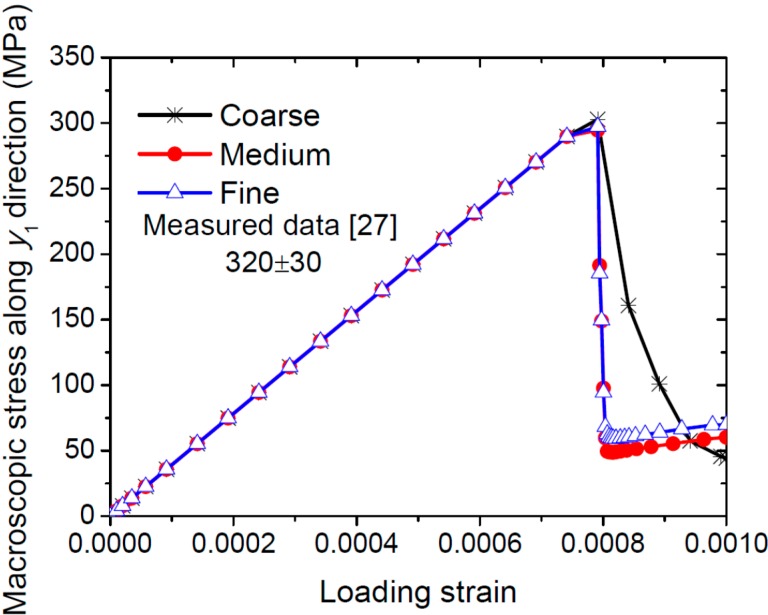
Influence of the mesh size on the macroscopic response of C/SiC composites subject to uniaxial tension along *y*_1_ direction.

The three meshes are employed to simulate the macroscopic uniaxial tension tests along *y*_1_ axis. The macroscopic elastoplastic responses of FRCs with perfect interfaces are considered here. Figure 4 shows the macroscopic stress-strain curves obtained with the three meshes. The medium and fine meshes predict the same tensile macroscopic $\sigma_{1}^{c}-\varepsilon_{x}^{c}$ curves, whereas the coarse mesh overestimates the $\sigma_{1}^{c}-\varepsilon_{1}^{c}$ curve. Thus, no improvement seems to come from the use of a mesh finer than the medium one. The medium mesh will be used in all subsequent simulations. Figure 4 also indicates a good agreement between the FE predictions and the experimental results measured by Heredia *et al.* [27]. They measured the transverse ultimate tensile strength of C/SiC composites with 22% carbon fibers as 320 ± 30 MPa.

### 2.4. Influence of the Interfacial Properties on the Macroscopic Strength

As mentioned below, the HBCs are less time consuming than PBCs, hence they are more suitable for sufficiently large RVEs or nonlinear analyses. In this subsection, HBCs are chosen to predict the macroscopically ultimate strengths of the composites in order to save the computational time. If we assume the fiber volume fraction to be constant, composite structures may vary their stiffness and strength due to damage accumulation such as matrix cracking and fiber breakage during the loading process of the composite members.

We define the initial damage traction dimensionless parameter $\tilde{t}=t_{\text{interface}}/f_{t}$, where *t_nn_* = *t_ss_* = *t_tt_* = *t*_interface_. The interface fracture energy, *G*_interface_, as an interfacial property, is selected to define the evolution of debonding in terms of the energy required for failure after the initiation of debonding. As depicted in Section 3.3, the fracture energy *G_c_* is equal to the area under the traction-separation curve, *i.e.*, it must be larger than ${\left(t_{\text{interface}}\right)}^{2}/\left(2K_{\text{interface}}\right)$. So we introduce an interfacial critical fracture energy dimensionless parameter $\tilde{G}=G_{\text{interface}}/\left({t_{\text{interface}}}^{2}/K_{\text{interface}}\right)$ to represent the relative fracture energy. Since the *t*_interface_ and *K*_interface_ are varied in the simulations, for simplicity, we define the critical fracture energy dimensionless parameter as $\tilde{G}=G_{\text{interface}}/\left[{\left(0.1f_{t}\right)}^{2}/0.1\frac{\left(E_{m}+E_{f}\right)}{2h_{\text{interface}}}\right]$.

#### 2.4.1. Uniaxial Transverse Tensile Strength along y_1_ Direction

**Figure 5:**
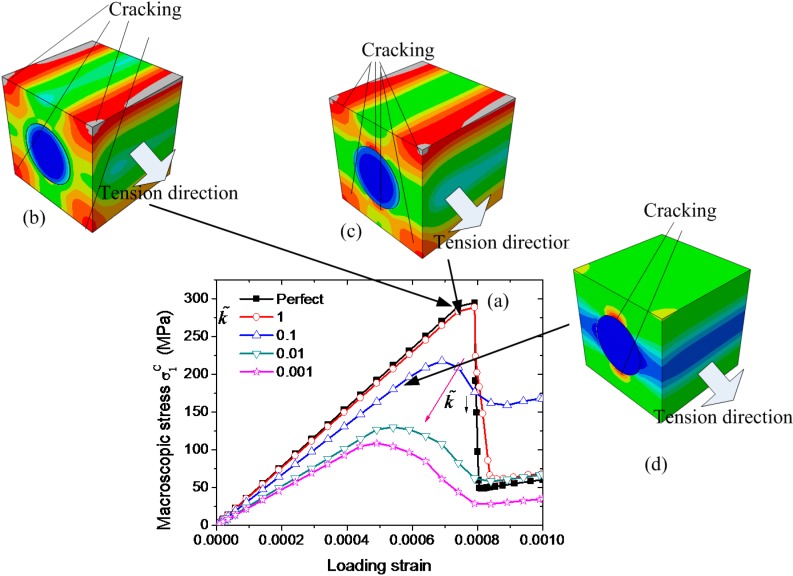
(**a**) Simulated macroscopic stresses with respect to the loading strain for the C/SiC composites with different interfacial stiffness subject to transverse tension along *y*_1 s_direction; inset (**b**) cracking near the interface for a weak interfacial stiffness; (**c**) cracking near the corners for a perfectly bonded interface between the fiber and the ceramics; and (**d**) cracking near the interface and the corners almost at the same time. The black arrows point different loading strain, and the red arrow represents the descent direction of $\tilde{k}$.

The uniaxial transverse tension along *y*_1_ direction is simulated by using the present HBC models. The relations of the macroscopic stresses $\sigma_{1}^{c}$ with the loading strain for the C/SiC composites with different interfacial stiffness are plotted in Figure 5a, where the critical interfacial damage strength and the critical interfacial fracture energy are assumed to be constant, *i.e.*, $\tilde{t}=0.1$ and $\tilde{G}$ = 100. The singularity associated with the FE modeling is inevitable at the interface between the fiber and the matrix or the boundaries of the RVE. It must be noted that the Drucker-Prager model is a “smeared crack model”, since it does not describe a single crack, but rather associates to any integration point with degraded mechanical properties. So the singularity affects slightly the overall response of the composites. It can be seen from Figure 5a that the interfacial stiffness plays an important role on the uniaxial transverse tensile strength. If we assume that the thickness of the interface is approximately one tenth of the fiber radius, the interface effect can be ignored when the interfacial modulus is stronger than the average of the Young’s moduli of the fiber and the matrix. As the interfacial stiffness decreases, the transverse tensile strength decreases significantly. The FE results show a difference in the damage onset in the matrix of the composites with different interfaces. For a perfect interface, the present model predicts the initiation of the damage in four regions near the corner of the RVE, as shown in Figure 5b. For a strong interface, the damage commences near both the corner and the interface, as shown in Figure 5c, while for a weak interface, it commences only near the interface as shown in Figure 5d.

**Figure 6:**
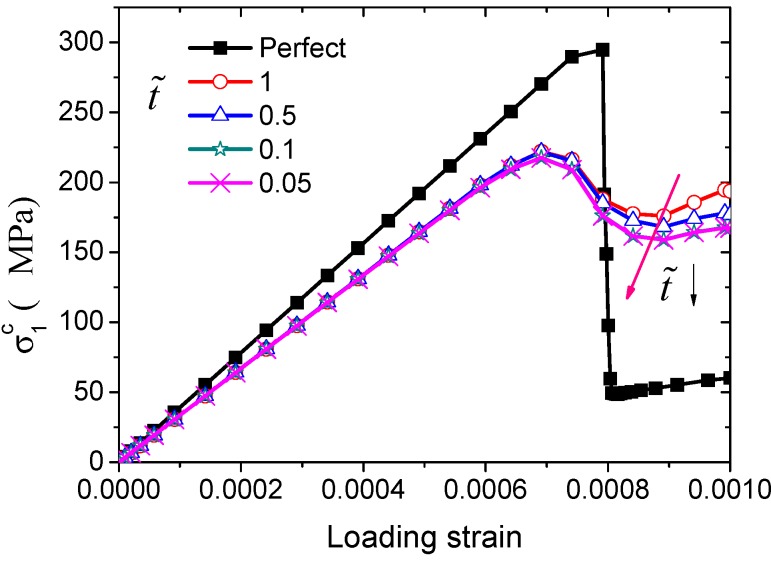
Simulated macroscopic stresses with respect to the loading strain for the C/SiC composites with different interfacial strengths subject to transverse tension along the *y*_1_ direction. Both the black and red arrows represent the descent direction of $\tilde{t}$.

**Figure 7:**
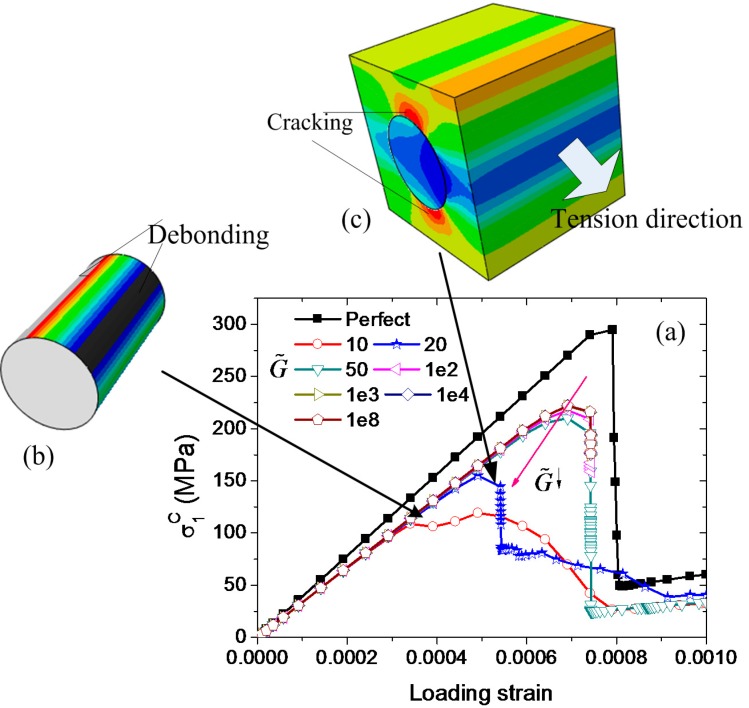
(**a**) Simulated macroscopic stresses with respect to the loading strain for the C/SiC composites with different interfacial fracture energy subject to transverse tension along *y*_1_ direction; inset (**b**) debonding on the interface; and (**c**) cracking near the interface in the matrix. The black arrows point different loading strain, and the red arrow represents the descent direction of $\tilde{G}$.

The influence of the interfacial strength on the ultimate transverse tensile strength is shown in Figure 6 for different $\tilde{t}$ where $\tilde{k}$ = 0.1 and $\tilde{G}=100$, It can be seen that the interfacial strength is not the major factor in determining the ultimate transverse tensile strength of the fiber-reinforced ceramic matrix composites. In the calculation, interfaces with different interfacial fracture energy are also considered. Figure 7a shows that the ultimate transverse tensile strength is very sensitive to the interfacial fracture energy when $\tilde{G}$ is lower than 50. We find that the composite will damage first due to the debonding of the interfaces (seen in Figure 7b) and then begin cracking near the interface in the ceramic matrix (seen in Figure 7c).

The ultimate transverse tensile strength is analytically given as [28]:

(1) $${\left({\sigma^{T}}_{1}\right)}_{\text{max}}=E_{1}{\left(\varepsilon_{1}^{T}\right)}_{\text{max}}$$

**Figure 8:**
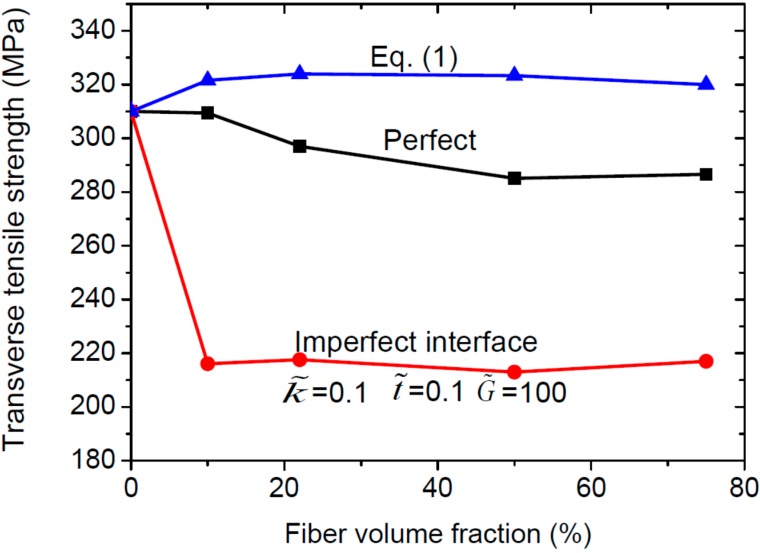
Ultimate transverse tensile strength with respect to the fiber volume fraction.

The ultimate tensile strain of composites ${\left(\varepsilon_{1}^{T}\right)}_{\text{max}}$ can be expressed in terms of ${\left(\varepsilon_{m}^{T}\right)}_{\text{max}}$ as,

(2) $${\left(\varepsilon_{1}^{T}\right)}_{\text{max}}=\left[\frac{2r}{s}\frac{E_{m}}{E_{f}}+\left(1-\frac{2r}{s}\right)\right]{\left(\varepsilon_{m}^{T}\right)}_{\text{max}}$$

where the overall transverse Young’s modulus is $E_{1}$ which can be found in Table 1, *s* represents the distance between centre of fibers, *r* is the radius of fibers, and ${\left(\varepsilon_{m}^{T}\right)}_{\text{max}}$ is the tensile failure strain of matrix. Figure 8 shows that the ultimate transverse tensile strength of C/SiC composites with perfect or imperfect interfaces is insensitive to the fiber volume fraction, and the imperfect interface may reduce the strength enormously. When the fiber volume fraction is very low (1.2% in our simulations), it can be seen from Figure 8 that those three values converge to one certain value (*i.e.*, the tensile strength of the matrix), implying that the interface effect can be ignored only when the fiber volume fraction is very low.

#### 2.4.2. Uniaxial Longitude Tensile Strength along y_3_ Direction

**Figure 9:**
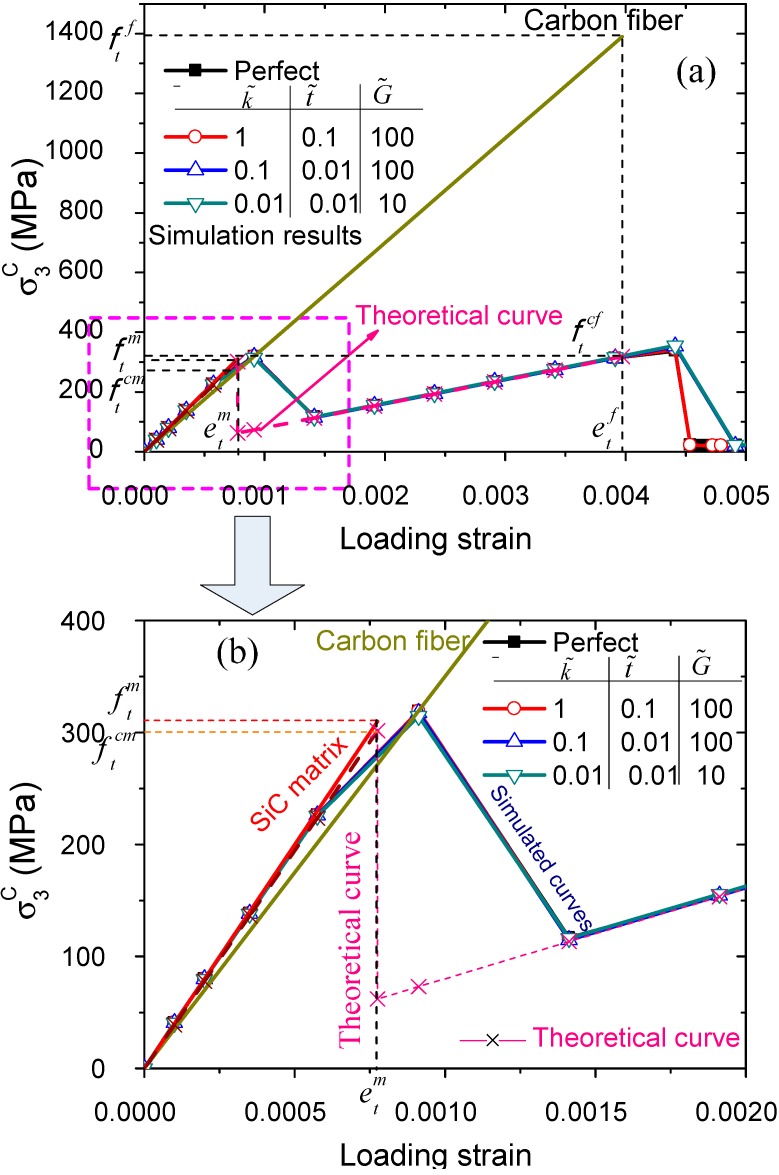
(**a**) Simulated macroscopic stresses in respect to the loading strain for the C/SiC composites with different interfacial properties subject to longitudinal tension along the *y*_3_ direction; and (**b**) the enlarged view in the range of loading strain 0.0 to 0.002.

The uniaxial longitude tension along y_3_ direction is simulated by using the present HBC model. The relations of the macroscopic stresses $\sigma_{3}^{c}$ with the loading strain for the C/SiC composites, considering imperfect interfaces with different interfacial properties, are plotted in Figure 9. It can be found that the longitudinal tensile strength of the C/SiC composites is almost independent on the interfacial properties. The composites with a weaker interface have a bit higher longitudinal tensile strength because the weaker interface inhibits the interaction of the brittle matrix and the fiber.

The fiber tensile strength is expressed as $f_{t}^{f}=E_{f}e_{t}^{f}$, and the matrix tensile strength is expressed as $f_{t}^{m}=E_{m}e_{t}^{m}$. The composite tensile stress $\sigma_{3}^{c}$ can be written as the function of the loading strain *e* as:

(3) $$\sigma_{3}^{c}=\{\begin{array}{c} \left[E_{f}V_{f}+E_{m}\left(1-V_{f}\right)\right]e\;e\leq e_{t}^{m}\\ \;V_{f}E_{f}e\;e_{t}^{m}<e\leq e_{t}^{f} \end{array}$$

As the loading strain *e* increases until $e_{t}^{m}$, the composite tensile stress increases and then drops sharply because of the crack of the ceramic matrix when *e* equals $e_{t}^{m}$. The corresponding failure strength is $f_{t}^{cm}=\left[E_{f}V_{f}+E_{m}\left(1-V_{f}\right)\right]e_{t}^{m}$), as shown in Figure 9a,b. After the matrix fails, only the fibers of the composite are subjected to the loading, so the composite’s ultimate longitudinal tensile strength is $f_{t}^{cf}=V_{f}E_{f}e_{t}^{f}$, as shown in Figure 9a. From our theoretical and simulated curves shown in Figure 9 it can be seen that the matrix fractures first and then the carbon fiber fractures, because carbon fiber’s failure strain $e_{t}^{f}$ is greater than SiC failure strain $e_{t}^{m}$. The comparison shows a great agreement between the simulated results and the theoretical curve.

**Figure 10:**
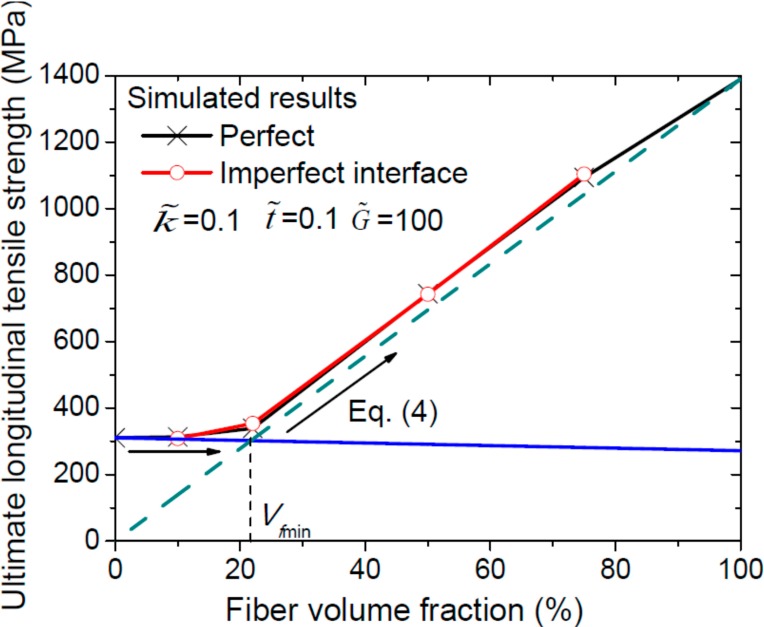
Ultimate longitudinal tensile strengths with respect to the fiber volume fraction. The arrow represents the ultimate longitudinal tensile strength with respect to the fiber volume fraction calculated using Equation (4).

For C/SiC composites having fiber failure strain greater than matrix failure strain, the variation of composite longitudinal tensile strength with fiber volume fraction is governed by:

(4) $$f_{t}^{c}=\{\begin{array}{c} E_{f}\frac{f_{t}^{m}}{E_{m}}V_{f}+f_{t}^{m}\left(1-V_{f}\right)\;0\leq V_{f}\leq V_{f\;\text{min}}\\ \;V_{f}f_{t}^{f}\;V_{f\;\text{min}}\;<V_{f}\leq 1 \end{array}$$

where $V_{f\;\text{min}}$ is the minimum fiber volume fraction below which by adding the fibers to the matrix, the C/SiC will have lower ultimate longitude tensile strength than the matrix. Figure 10 shows that as the fiber volume fraction increases, the ultimate longitude tensile strength of C/SiC composites increases sharply, which satisfies the mixture rule.

## 3. Experimental Section

### 3.1. Homogenized 3D RVE for FRCs

**Figure 11:**
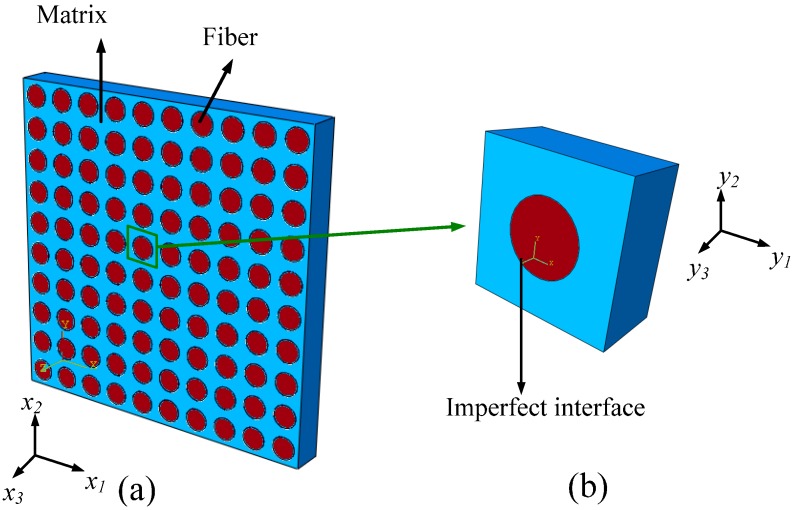
(**a**) Sketch of fiber-reinforced ceramic matrix composites; and (**b**) an enlarged 3D unit cell.

In the assumption of the two-scale asymptotic homogenization method, unidirectional and long-fiber composites are simplified as composites constructed by periodically and uniformly distributed unit cells, as shown in Figure 11a. An enlarged unit cell (also called RVE) is shown in Figure 11b. Making use of mathematical homogenization, a linear elastic static problem with periodic conditions could be decomposed into uncoupled fine and coarse scale problems. The macroscopically effective mechanical properties of the FRCs could be determined through the estimation of one unit cell at fine scale using the perturbation technology. The computational homogenization approach for linear and nonlinear solid problems, presented in [29] is used in our analysis.

For coarse scale problem we have:

(5) $${\bar{L}}_{ijmn}\varepsilon_{mn,x_{j}}^{c}+{\bar{b}}_{i}=0\;\text{on}\;\Omega$$

(6) $$u_{i}^{c}\left(x\right)={\bar{u}}_{i}\;\text{on}\Gamma_{u};{\bar{\sigma }}_{ij}n_{j}={\bar{t}}_{i}\;\text{on}\;\Gamma_{t}$$

where Ω is the domain of the coarse scale problem; $\Gamma_{u}$ and $\Gamma_{t}$ the displacement and traction boundaries, respectively; **x** is the coarse scale position vectors, respectively; ζ satisfies 0<ζ≤1; $u_{i}^{c}$ is the coarse scale displacement as a function of **x**; $\varepsilon_{mn}^{c}=\frac{1}{2}\left(\frac{\partial u_{m}^{c}}{\partial x_{n}}+\frac{\partial u_{n}^{c}}{\partial x_{m}}\right)$ is the coarse scale strain, and ${\bar{b}}_{i}$ is the average unit cell body force; ${\bar{u}}_{i}$, ${\bar{b}}_{i}$ and ${\bar{\sigma }}_{ij}$ represent displacement, traction and stress boundary conditions, respectively. Summation convention is employed for repeated indices.

For unit cell problem,

(7) $${\left[L_{ijkl}\left(\chi_{\left(k,y_{l}\right)mn}+I_{klmn}\right)\right]}_{,y}=0\;\text{on}\;\Theta$$

(8) $$\chi_{imn}\left(y\right)=\chi_{imn}\left(y+Y\right)\text{on}\;\partial \Theta ;\;\chi_{imn}\left(y\right)=0\;\text{on}\;\partial \Theta^{\text{vert}}$$

where Θ is the domain of the unit cell; $\partial \Theta^{\text{vert}}$ the vertices of the unit cell; y=x/ζ the fine scale position vector; **Y** the period of the associated function; $I_{klmn}=\left(\delta_{mk}\delta_{nl}+\delta_{nk}\delta_{ml}\right)$; $\chi_{\left(k,y_{l}\right)mn}=\frac{1}{2}\left(\frac{\partial \chi_{kmn}}{\partial y_{l}}+\frac{\partial \chi_{lmn}}{\partial y_{k}}\right)$; and the homogenized constitutive tensor ${\bar{L}}_{ijmn}$ is given as:

(9) $${\bar{L}}_{ijmn}=\frac{1}{\left|\Theta \right|}\int_{\Theta }\sigma_{ij}^{mn}\left(y\right)d\Theta$$

where $\sigma_{ij}^{mn}\left(y\right)$ is the stress influence function defined as:

(10) $$\sigma_{ij}^{mn}\left(y\right)=L_{ijkl}\left(\chi_{\left(k,y_{l}\right)mn}+I_{klmn}\right)$$

The two-scale algorithm described here can be generalized to account for material and geometric nonlinearities.

**Figure 12:**
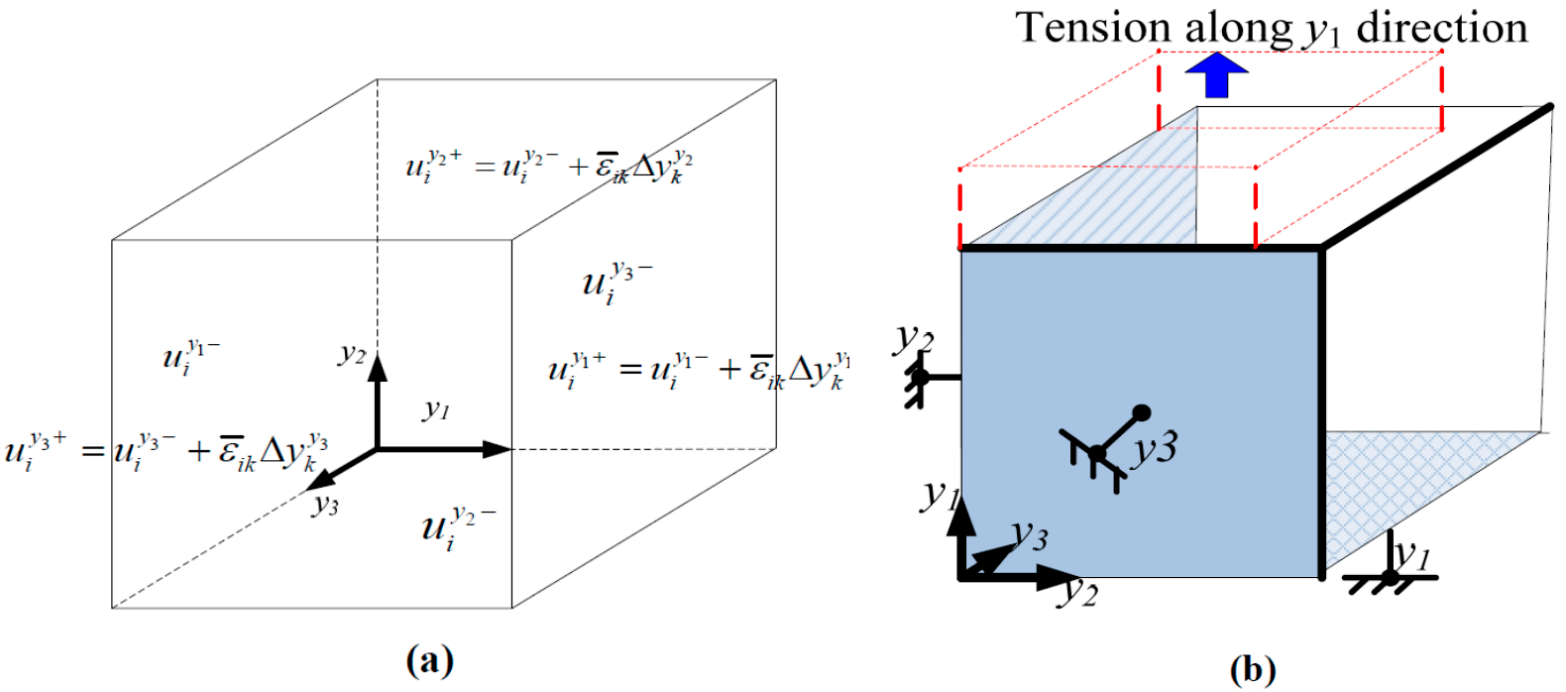
(**a**) An 3D RVE with periodic boundary conditions (PBCs); and (**b**) homogenization boundary conditions (HBCs)―tensile case along *y*_1_ direction (only the normal directions are fixed at the boundaries). The blue arrow represents the tension direction, and the red frame represents the configuration after tension.

It is noted that boundary conditions can significantly affect the macro behavior of the RVEs during the homogenization simulation process. To study the effect, two types of boundary conditions are generally used. If an RVE with 3D periodic boundary conditions (PBCs) is used, the simulation results represent a macro structure consisting of periodically repeated cells. While choosing 3D homogeneous boundary conditions (HBCs), the simulation results would consider the RVE as the macro structure itself with its micro-constituents. HBCs are less time-consuming in computation than periodic boundary conditions, hence they are more suitable for sufficiently large RVEs. In the simulation, both PBCs and HBCs are chosen to estimate macroscopically effective elastic parameters of the FRCs (as shown in Figure 12a), while HBCs are chosen to predict macroscopically ultimate strengths in order to save computational time (as shown in Figure 12b).

### 3.2. Constitutive Model for the Components

Unidirectional long fiber-reinforced ceramic matrix composites are investigated in the present study. The fibers are supposed to be linear elastic when the stress level is below its tensile strength. The brittle behavior of the ceramics is described using the Drucker-Prager yield criterion which has already been implemented into the commercial FE software ABAQUS [30]. This model was originally developed for plain concrete subjected to multiaxial stresses and has been successfully used to estimate the transverse strengths for the ceramic matrix composites [20].

**Figure 13:**
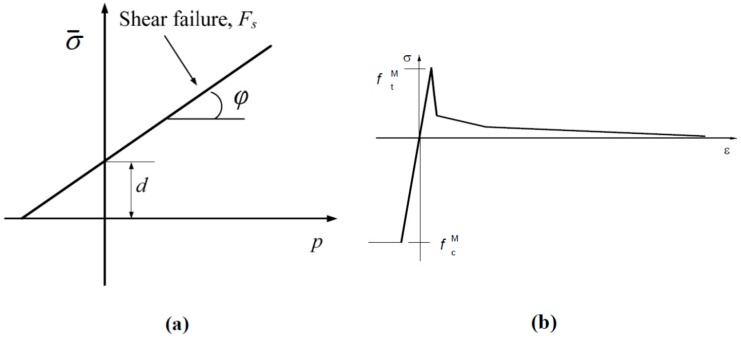
(**a**) Yield surfaces in the *p*-$\bar{\text{σ}}$ plane in the Drucker-Prager model; and (**b**) uniaxial stress-strain curve.

The Drucker-Prager failure surface is given by:

Fs = σ^–^–p tan φ–d = 0 (11)

where φ is the material’s angle of friction and *d* is its cohesion (see Figure 13a). The equivalent compressive stress *p* is expressed as a function of the principal stresses $\sigma_{1},\sigma_{2}$ and $\sigma_{3}$:

(12) $$p=-\frac{1}{3}\left(\sigma_{1}+\sigma_{2}+\sigma_{3}\right)$$

Here we denote $\bar{\sigma }$ as the Mises equivalent stress:

(13) $$\bar{\sigma }=\sqrt{\frac{1}{2}\left[{\left(\sigma_{1}-\sigma_{2}\right)}^{2}+{\left(\sigma_{2}-\sigma_{3}\right)}^{2}+{\left(\sigma_{3}-\sigma_{1}\right)}^{2}\right]}$$

We can determine the material’s angle of friction φ and its cohesion *d* through the uniaxial tensile strength $f_{t}$ and uniaxial compressive strength $f_{c}$:

(14) $$\text{tan}\;\varphi =\frac{3\left(f_{c}-f_{t}\right)}{f_{c}+f_{d}}$$

(15) $$d=\frac{2f_{t}f_{c}}{f_{t}+f_{c}}$$

The cohesion *d* is equal to yield stress in the case of *f*_c_ = *f*_t_, *i.e.*, no difference between compressive and tensile strengths. For SiC ceramics, the maximum tensile strength *f*_t_ and maximum compressive strength *f*_c_ are 310 and 3900 MPa, respectively. Making use of Equations (14) and (15), the calculated material’s angle of friction φ = 68.6°, dilation angle θ = φ = 68.6° which satisfies the associate flow. A sharp post-peak drop in strength is defined for approaching the behavior of a perfectly brittle ceramic material, as shown in Figure 13b. The post-peak strain softening behavior of the ceramics is inputted in the Drucker-Prager model, and can be simulated by means of the modified Riks method in ABAQUS. For carbon fiber, perfectly elastic behavior and a tensile strength of 1390 MPa is assumed. If the stress level exceeds the maximum strength for matrix and/or fiber, the Young’s modulus *E* is degraded to 1% from its initial value at a particular integration point, while the shear modulus *G* is reduced to 20% of the initial value under the assumption that some shear stiffness remains due to the friction still present on the failure plane [31], which is realized through the user subroutine USDFLD in ABAQUS. The material properties used in the simulations are listed in Table 2. The superscripts *m* and *f* appearing in Table 1 and afterwards represent the variables associated with the matrix and fiber, respectively. 

**Table 2:** Properties of the component materials of C/SiC composites.

Material Properties	SiC	Carbon-Fiber
Young’s modulus (GPa)	*E*_m_ 400	*E*_f_ 350
Poisson’s ratio	*v*_m_ 0.14	*v*_f_ 0.3
Tensile strength (MPa)	$f_{t}^{m}$ 310	$f_{t}^{f}$ 1380
Compressive strength (MPa)	$f_{c}^{m}$ 3900	-
Volume fraction	*V*_m_ 0.78	$V_{f}$ 0.22

### 3.3. Cohesive Interfacial Model for Fiber/Matrix Interfaces

**Figure 14:**
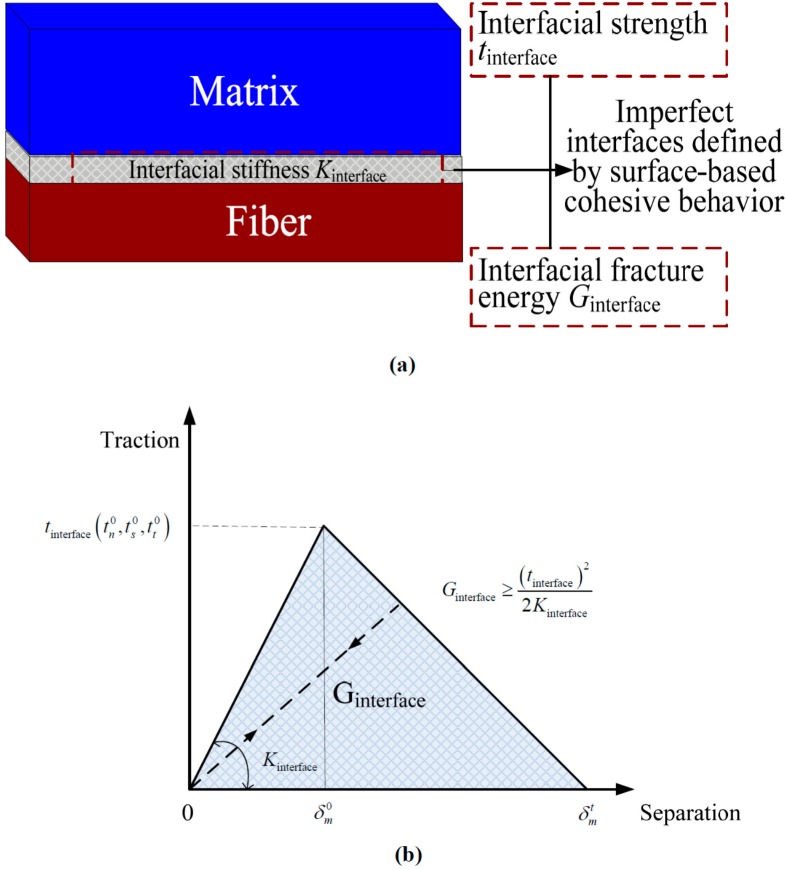
(**a**) Imperfect interface defined by surface-based cohesive behavior; and (**b**) typical traction-separation response.

The cohesive elements employ failure criteria that combine aspects of strength-based analysis to predict the onset of the softening process at the interface and fracture mechanics to predict debonding propagation. If the interface thickness is negligibly small, it can be straightforward to define the surface-based cohesive response of the cohesive layer directly in terms of traction *versus* separation (see Figure 14a), which will spend less computational time compared with the cohesive element in ABAQUS. The available traction-separation model in ABAQUS assumes initially linear elastic behavior followed by the initiation and evolution of damage.

The nominal traction stress vector, *t*, consists of three-components: *t*_n_, *t*_s_ and *t*_t_, which represent the normal (along the local 3-direction) and the two shear tractions (along the local 1- and 2-directions), respectively. The corresponding separations are denoted by δ*_n_*, δ*_s_* and δ*_t_*. Denoting *T*_0_ as the original thickness of the cohesive element, the nominal strains can be defined as:

(16) $$\varepsilon_{n}=\frac{\delta_{n}}{T_{0}},\varepsilon_{s}=\frac{\delta_{s}}{T_{0}},\varepsilon_{t}=\frac{\delta_{t}}{T_{0}}$$

The elastic behavior can then be written as:

(17) $$t=\left\{\begin{array}{l} t_{n}\\ t_{s}\\ t_{t} \end{array}\right\}=\left[\begin{array}{ccc} K_{nn} & K_{ns} & K_{nt}\\ K_{sn} & K_{ss} & K_{st}\\ K_{tn} & K_{ts} & K_{tt} \end{array}\right]\left\{\begin{array}{l} \delta_{n}\\ \delta_{s}\\ \delta_{t} \end{array}\right\}$$

Damage of the traction-separation response for cohesive surface is defined within the same general framework used for conventional materials [32]. A quadratic stress damage initiation criterion and an energy damage evolution law are defined for modeling the debonding of the interfaces, as shown in Figure 14b. The quadratic stress criterion suggests that damage initiates when a quadratic interaction function involving the contact stress ratios reaches the value of one. This criterion can be represented as:

(18) $${\left\{\frac{\langle t_{n}\rangle }{t_{n}^{0}}\right\}}^{2}+{\left\{\frac{t_{s}}{t_{s}^{0}}\right\}}^{2}+{\left\{\frac{t_{t}}{t_{t}^{0}}\right\}}^{2}=1$$

where *t*_n_, *t*_s_, and *t*_t_ represent the contact stress normal to the interface, along the first and the second shear directions, respectively $t_{n}^{0}$, $t_{s}^{0}$ and $t_{t}^{0}$ represent the peak values of the contact stress when the separation is either purely normal to the interface or purely in the first or the second shear direction, respectively. The symbol 〈 〉 represents the Macaulay bracket with the usual interpretation, indicating that a purely compressive stress state does not initiate damage. In the damage evolution criterion, damage evolution can be defined based on the energy that is dissipated as a result of the damage process, also called the fracture energy. The fracture energy $G_{c}$ is equal to the area under the traction-separation curve shown in Figure 14b.

## 4. Conclusions

Based on the homogenization method with periodic or homogenization boundary conditions, a 3D RVE model is developed. The proposed model has been validated with the theoretical results for the composites with perfect bonding between the fiber and the matrix. From the study, we found that for composites with imperfect interfaces, as the interface stiffness decreases, the *E*_1_, *G*_12_ and *G*_23_ decrease, but *E*_3_ and *v*_23_ remain almost as constants.

The obtained numerical results for the transverse tensile strength of the composites with perfect bonding agree well with those from experiments.

The imperfect interfaces between the fiber and the matrix are taken into account by introducing cohesive contact surfaces. The influences of the interface on the elastic constants and the tensile strengths are examined using the interfacial model. It is found that the imperfect interface can induce different damage onset in the matrix of the composites with different interfacial properties. In contrast the interfacial strength, the interfacial stiffness and fracture energy can significantly influence the transverse tensile strength of the composites, while the longitudinal tensile strength of the composites is almost independent of the interfacial properties, and it increases sharply with an increase in the fiber volume fraction, satisfying the mixture rule. The results indicate that the proposed approach is simple to use and efficient for performing realistic numerical analyses on complex 3-D fiber-reinforced composites.
